# Quantum Two Player Game in Thermal Environment

**DOI:** 10.1371/journal.pone.0134916

**Published:** 2015-08-31

**Authors:** Jerzy Dajka, Dawid Kłoda, Marcin Łobejko, Jan Sładkowski

**Affiliations:** 1 Institute of Physics, University of Silesia, Katowice, Poland; 2 Silesian Center for Education and Interdisciplinary Research, University of Silesia, Chorzów, Poland; University of Nottingham, UNITED KINGDOM

## Abstract

A two-player quantum game is considered in the presence of thermal decoherence. It is shown how the thermal environment modeled in terms of rigorous Davies approach affects payoffs of the players. The conditions for either beneficial or pernicious effect of decoherence are identified. The general considerations are exemplified by the quantum version of Prisoner Dilemma.

## Introduction

Information processing is a physical phenomenon and therefore information theory is inseparable from both applied and fundamental physics. Attention to the quantum aspects of information processing revealed new perspectives in computation, cryptography and communication methods. In numerous cases a quantum description of the system provides some advantages over the classical situation, at least in theory. Does quantum mechanics offer more subtle mechanisms of playing games? In game theory one often has to consider strategies that are probabilistic mixtures of pure strategies [1, 2]. Can they be intertwined in a more complicated way by exploring interference or entanglement? There certainly are situations in which quantum theory can enlarge the set of possible strategies [3–5]. This is a very nontrivial issue as genuine quantum systems usually are unstable and their preparation and maintenance might be difficult e.g. due to decoherence [6–10]. Note that quantum formalism can be used in game theory in a more abstract way without any reference to physical quantum states [11–13]—the decoherence is not a problem in such approaches. The question is if quantum games are of any practical value. The answer is positive and some commercial cryptographical and communication methods/products are already available. The field of quantum auctions seems to be promising too [14, 15]. In this paper we would like to show how the decoherence in quantum games can be described in terms of completely positive Davies maps [16]. This should be compared with approaches presented in [6–10, 17]. We focus our attention on the quantum Prisoner Dilemma [4] but the approach can be used in other games too. We show that properly utilized decoherence can, sometimes and at certain circumstances, have a beneficial effect. The paper is organized as follows. We will begin by a brief presentation of quantum game formalism. Then we will describe our approach to the decoherence in quantum games. Finally we will discuss some problems that should be addressed in the near future.

## Methods

### Quantum game

The general definition of a quantum game would be involved. Here by a quantum game we understand a quantum system that can be manipulated by at least one party and for which utilities of moves can be reasonably defined. We shall suppose that all players know the state of the game at the beginning and, possibly, at some crucial stages of the actual game being played. We neglect the possible technical problems with actual identification of the state. Implementation of a quantum game should include measuring apparatuses and information channels that provide necessary information on the state of the game at crucial stages and specify the moment and methods of its termination. We will not discuss these issues here.

We will consider only two–player quantum games: the generalization for the N players case is straightforward. Therefore we will suppose that a two–player quantum game Γ = (𝓗, *ρ*_*i*_, *S*_*A*_, *S*_*B*_, *P*_*A*_, *P*_*B*_) is completely specified by the underlying Hilbert space 𝓗 of the quantum system [18], the initial state given by the density matrix *ρ*_*i*_ ∈ 𝓢(𝓗), where 𝓢(𝓗) is the associated state space, the sets *S*_*A*_ and *S*_*B*_ of quantum operations representing moves (strategies) of the players, and the pay–off (utility) functions *P*_*A*_ and *P*_*B*_, which specify the pay–off for each player after the final measurement is performed on the final state *ρ*_*f*_. A quantum strategy *s*_*A*_ ∈ *S*_*A*_, *s*_*B*_ ∈ *S*_*B*_ is a collection of admissible quantum operations, that is the mappings of the space of states onto itself. One usually supposes that they are completely positive trace–preserving maps. Schematically we have:

$$\begin{array}{c} \rho_{i}\mapsto \left(s_{A},s_{B}\right)\mapsto \rho_{f}\mapsto \text{measurement}\Rightarrow \left(P_{A},P_{B}\right)\;. \end{array}$$

This scheme for a quantum two–player game can be implemented as a quantum map:

$$\begin{array}{ccc} \rho_{f} & = & J^{-1}\circ S\circ D\circ J\left(\rho_{i}\right), \end{array}$$ (1)

where initially

$$\begin{array}{ccc} \rho_{i} & = & \left|00\rangle \langle 00\right| \end{array}$$ (2)

describes identical starting positions of Alice (*A*) and Bob (*B*). Using entanglement is one of possible ways to utilize the power of quantum mechanics in quantum games. Here the state of players is transformed using

$$\begin{array}{ccc} J(\rho ) & = & J\left(\gamma \right)\rho J{\left(\gamma \right)}^{†} \end{array}$$ (3)

with

$$\begin{array}{ccc} J(\gamma ) & = & \cos\left(\gamma /2\right)I⊗I+i\sin\left(\gamma /2\right)\sigma_{x}⊗\sigma_{x}\; \end{array}$$ (4)

into an entangled state. Here 𝓘 and *σ*_*x*_ denote the identity operator and the Pauli matrix, respectively. Note that due to the presence of noise the amount of entanglement does not necessary increase with increase of *γ*. Due to omnipresent decoherence the entangled state of two players can be affected by thermal dissipation and dephasing described by completely positive Davies map 𝔻. Description of its detailed properties is postponed to the next section.

For a standard (canonical) matrix representation of quantum states of a two level system

$$\begin{array}{ccc} \left|0\rangle \langle 0\right| & \to & \left(\begin{array}{cc} 0 & 0\\ 0 & 1 \end{array}),\;\;\;\;|1\rangle \langle 1|\to (\begin{array}{cc} 1 & 0\\ 0 & 0 \end{array}\right) \end{array}$$ (5)

the initial state in Eq (2) is given by

$$\begin{array}{ccc} \rho_{i} & = & (\begin{array}{cccc} 0 & 0 & 0 & 0\\ 0 & 0 & 0 & 0\\ 0 & 0 & 0 & 0\\ 0 & 0 & 0 & 1 \end{array}), \end{array}$$ (6)

whereas the entangling operator in Eq (4) reads as

$$\begin{array}{ccc} J(\gamma ) & = & (\begin{array}{cccc} \cos(\gamma /2) & 0 & 0 & i\sin(\gamma /2)\\ 0 & \cos(\gamma /2) & i\sin(\gamma /2) & 0\\ 0 & i\sin(\gamma /2) & \cos(\gamma /2) & 0\\ i\sin(\gamma /2) & 0 & 0 & \cos(\gamma /2) \end{array}). \end{array}$$ (7)

Then Eq (3) has, for the initial state given by Eq (2), the following matrix form [19]:

$$\begin{array}{ccc} J(\rho_{i}) & = & (\begin{array}{cccc} \sin{\left(\gamma /2\right)}^{2} & 0 & 0 & i\cos(\gamma /2)\sin(\gamma /2)\\ 0 & 0 & 0 & 0\\ 0 & 0 & 0 & 0\\ -i\cos(\gamma /2)\sin(\gamma /2) & 0 & 0 & \cos{\left(\gamma /2\right)}^{2} \end{array}). \end{array}$$ (8)

The individual strategies of players *S*_*X*_, *X* = *A*, *B* are implemented as follows:

$$\begin{array}{c} S\left(\rho \right)=\left(S_{A}⊗S_{B}\right)\rho {\left(S_{A}⊗S_{B}\right)}^{†}. \end{array}$$ (9)

In this work we assume that there are only two classical pure strategies available, identity and flip operation:

$$\begin{array}{ccc} S_{A} & \in & \{I,F\equiv i\sigma_{x}\}. \end{array}$$ (10)

We also allow Bob to follow his strategy by a pure quantum strategy i.e.

$$\begin{array}{ccc} S_{B} & \in & \left\{IU,FU\right\} \end{array}$$ (11)

where the quantum strategy is given by unitary transformation with the explicit matrix form [19]

$$\begin{array}{ccc} U(\theta ,\alpha ,\beta ) & = & (\begin{array}{cc} e^{i\alpha }\cos\left(\theta /2\right) & ie^{i\beta }\sin\left(\theta /2\right)\\ ie^{-i\beta }\sin\left(\theta /2\right) & e^{-i\alpha }\cos\left(\theta /2\right) \end{array}). \end{array}$$ (12)

In other words, we assume that the pure strategies of both players differ because only one of them (Bob) recognizes that information is stored in qubits rather than bits i.e. Bob can utilize richer class of operations formalized by *U*. This knowledge is beneficial provided that Bob can influence Alice’s strategy. It is the case as the 𝕁(*γ*) in Eq (4) entangles Alice’s and Bob’s systems for *γ* ≠ 0. Let us emphasize that in the presence of the entanglement, the actions of the players are not fully independent, as their qubits remain correlated. In real systems the correlation is never maximal due to the omnipresent decoherence affecting qubits represented in Eq (1) by 𝔻. It is assumed here that the decoherence influences players in the time when they are selecting their strategies 𝕊. It is clear that in general decoherence affects quantum states used in the game at any stage of its time evolution. However, if a considered time interval (e.g. between preparation of the initial state Eq (2) and applying entangling operator 𝕁) is significantly shorter than the time scale of decoherence process one can safely neglect any dissipation of information in that time interval. Physically, we assume that the only time interval which is comparable (or larger) to the decoherence time scale is the one which is required by the players to work out their strategies. We ask then how the quantum game becomes modified for given model of quantum dissipation.

### The model of decoherence

The only fully natural source of decoherence affecting quantum systems is due to their environment causing both energy and information dissipation. For our model considerations we assume that at least one of two qubits in the state *χ*_*AB*_ = 𝕁(*ρ*_*i*_) shared by Alice and Bob just before applying their strategies 𝕊_*A*, *B*_ interacts with its own environments *E*_*A*, *B*_. As Alice and Bob can be separated from each other we neglect any direct interaction both between their qubits (via proper Hamiltonian term) and the environments *E*_*A*, *B*_. In other words, Hamiltonian of the total system is simplified to the form:

$$\begin{array}{ccc} H & = & H_{A}+H_{B}+H_{AE_{A}}^{int}+H_{BE_{B}}^{int}. \end{array}$$ (13)

We also assume that qubits *A* and *B* are identical:

$$\begin{array}{ccc} H_{A} & = & H_{B}=\frac{\omega }{2}\left(|1\right\rangle \langle 1|-|0\rangle \left\langle 0|\right). \end{array}$$ (14)

Moreover, we assume that the interaction between qubits and their environments satisfies Davies weak coupling approach [20, 21]. Davies approach allows for a mathematically rigorous construction of a qubit’s reduced dynamics (with respect to environments) in terms of a completely positive (strictly Markovian) semigroup [20, 21]. Moreover, Davies semigroups can be rigorously and consistently derived from microscopic Hamiltonian models of open systems [20], so they satisfy most desired thermodynamic and statistical–mechanical properties such as the detailed balance condition [21]. Davies approximation has been successfully used in studies of various problems in quantum information and physics of open quantum systems including entanglement dynamics [22], quantum discord [23, 24] or properties of geometric phases of qubits [25] and thermodynamic properties of nano–systems [26]. Here, instead of exploring the full power of Davies semigroups, we consider only certain elements of Davies dynamical semigroups: Davies *maps*[16] which inherit all the properties proved to hold true for Davies semigroups with the complete positivity as the most desired among them. Here we adopt notation of Ref. [27] (instead of that used in Ref. [16]) and recapitulate an explicit form of Davies maps applied to the initial state given by Eq (8):

$$\begin{array}{c} \chi_{AB}\left(0\right)=J\left(\rho_{i}\right) \end{array}$$ (15)

and we consider three possibilities:

$$\begin{array}{ccc} \chi_{AB}\left(t\right) & = & \left[U_{A}⊗D_{B}\right]\chi_{AB}\left(0\right) \end{array}$$ (16)

$$\begin{array}{ccc} \chi_{AB}\left(t\right) & = & \left[D_{A}⊗U_{B}\right]\chi_{AB}\left(0\right) \end{array}$$ (17)

$$\begin{array}{ccc} \chi_{AB}\left(t\right) & = & \left[D_{A}⊗D_{B}\right]\chi_{AB}\left(0\right), \end{array}$$ (18)

where *U*_*A*, *B*_ denotes Hamiltonian dynamics of a noiseless qubit and the Davies map *D* = *D*_*A*, *B*_(*p*, *A*, *G*, *ω*, *t*) reads as follows [16]:

$$\begin{array}{ccc} D|1\rangle \langle 1| & = & \left[1-\left(1-p\right)\left(1-e^{-At}\right)\right]\left|1\rangle \langle 1\right|+\left(1-p\right)\left(1-e^{-At}\right)\left|0\rangle \langle 0\right| \end{array}$$ (19)

$$\begin{array}{ccc} D|1\rangle \langle 0| & = & e^{i\omega t-Gt}\left|1\rangle \langle 0\right| \end{array}$$ (20)

$$\begin{array}{ccc} D|0\rangle \langle 1| & = & e^{-i\omega t-Gt}\left|0\rangle \langle 1\right| \end{array}$$ (21)

$$\begin{array}{ccc} D|0\rangle \langle 0| & = & p\left(1-e^{-At}\right)\left|1\rangle \langle 1\right|+\left[1-\left(1-e^{-At}\right)p\right]|0\rangle \langle 0|, \end{array}$$ (22)

or, in terms of coherence vector formalism adopted in Ref. [16]:

$$\begin{array}{c} D=(\begin{array}{cccc} 1-(1-e^{-At})p & 0 & 0 & (1-e^{-At})p\\ 0 & e^{i\omega t-Gt} & 0 & 0\\ 0 & 0 & e^{-i\omega t-Gt} & 0\\ 1+\left(1-p\right)\left(1-e^{-At}\right) & 0 & 0 & 1-\left(1-p\right)\left(1-e^{-At}\right) \end{array}) \end{array}$$ (23)

where the matrix is acting on a density matrix in the column-vector representation, i.e.:

$$\begin{array}{c} \left(\begin{array}{cc} \rho_{00} & \rho_{01}\\ \rho_{10} & \rho_{11} \end{array})\to (\begin{array}{c} \rho_{00}\\ \rho_{01}\\ \rho_{10}\\ \rho_{11} \end{array}\right) \end{array}$$ (24)

Let us notice that, contrary to the Dirac bra–ket formalism which we adopt in this work, the coherence vector formalism is not very convenient for presenting states of composite qubit–qubit systems as it requires vectors with 16 elements and the two–qubit operators require (16 × 16)–dimensional matrices.

The *p* ∈ [0, 1/2] parameter appearing in transformation is related to the temperature (here we set *k*_*B*_ = 1) via:

$$\begin{array}{ccc} p & = & \exp(-\omega /2T)/[\exp(-\omega /2T)+\exp(\omega /2T)]. \end{array}$$ (25)

The parameters *A* = 1/*τ*_*R*_ and *G* = 1/*τ*_*D*_, if interpreted in terms of spin relaxation dynamics [28], are related to the energy relaxation time *τ*_*R*_ and the dephasing time *τ*_*D*_, respectively [16]. The parameters *A*, *G* and *p* depend solely on details of the qubit–environment coupling encoded in the Hamiltonian Eq 14 [21]. Fulfilling the inequalities [28]

$$\begin{array}{ccc} G & \geq & A/2\geq 0 \end{array}$$ (26)

guarantees that the Davies map is a trace-preserving completely positive map [21] as it is an element of the Davies semigroup which is proved to be completely positive and trace preserving [21]. This property allows one to apply Davies maps to any part of a composite system, also in the case when the subsystems are initially entangled. Let us notice that it is crucial as the decoherence 𝔻 in Eq (1) is a tensor product of two maps with at least one being the Davies map. Complete positivity guarantees that the ‘output’ *χ*_*AB*_(*t*) in Eq (16) is a quantum state. The limiting case *A* = 0 and *G* ≠ 0 corresponds to pure dephasing without dissipation of energy. The Davies decoherence introduces two parameters *A* and *G* modifying quantum game which we consider in addition to the ‘generic set’ consisting of the entangling parameter *γ* and three parameters constituting *U* in Eq (12).

### Payoffs

Payoff, the results of the game, can be calculated as an expectation value, weighted by certain game–dependent real numbers *a*, *b*, *c*, *d* constituting the payoff matrix:

Bob:0Bob:1Alice:0(a,a)(d,c)Alice:1(c,d)(b,b) (27)

which leads the the following pay-off operator

$$\begin{array}{c} {\$}_{A}=b\text{Tr}\left(|11\right\rangle \left\langle 11|\rho_{f}\right)+a\text{Tr}\left(|00\right\rangle \left\langle 00|\rho_{f}\right)+c\text{Tr}\left(|10\right\rangle \left\langle 10|\rho_{f}\right)+d\text{Tr}\left(|01\right\rangle \left\langle 01|\rho_{f}\right)\\ {\$}_{B}=b\text{Tr}\left(|11\right\rangle \left\langle 11|\rho_{f}\right)+a\text{Tr}\left(|00\right\rangle \left\langle 00|\rho_{f}\right)+d\text{Tr}\left(|10\right\rangle \left\langle 10|\rho_{f}\right)+c\text{Tr}\left(|01\right\rangle \left\langle 01|\rho_{f}\right). \end{array}$$ (28)

For example, the strategy profile (*S*_*A*_ = 0, *S*_*B*_ = 1) is encoded in the quantum state ∣01⟩ and results in payoffs *c* for Bob and *d* for Alice. The trace operation represents projective measurements performed on the output state.

We describe the influence of a thermal environment in terms of payoff’s differences

$$\begin{array}{ccc} \Delta \$ & = & {\$}_{B}-{\$}_{A} \end{array}$$ (29)

$$\begin{array}{ccc} \Delta {\$}_{B} & = & {\$}_{\tilde{B}}-{\$}_{A} \end{array}$$ (30)

$$\begin{array}{ccc} \Delta {\$}_{A} & = & {\$}_{B}-{\$}_{\tilde{A}} \end{array}$$ (31)

$$\begin{array}{ccc} \Delta {\$}_{AB} & = & {\$}_{\tilde{B}}-{\$}_{\tilde{A}} \end{array}$$ (32)

where the tilde denotes the “noisy” player. The signs of Δ’s in Eqs (29–32) identify the winner of the game i.e. the one of two players whose payoff is larger.

Below we present explicit formulas calculated for four typical quantum strategies. As the general formulas are very complicated we present the case *θ* = *π*/2 for simplicity. Let us notice that due to symmetry of the system the payoff differences do not depend on *a* and *b*. There is also no difference between the payoff of the ‘noisy’ player and that which is unaffected by the environment provided that there is no energy exchange between noisy qubit and the thermal bath i.e. *A* = 0.

Let us start with the considering the strategy profile (𝓘,𝓘*U*). The payoffs are given by the formulas:

$$\begin{array}{ccc} \Delta {\$}_{B} & = & \frac{1}{2}\left(c-d\right)\left[\cos^{2}\left(\gamma \right)+\frac{\cos\left(2\beta +2\omega t\right)\sin^{2}\left(\gamma \right)}{e^{Gt}}\right] \end{array}$$ (33)

$$\begin{array}{ccc} \Delta {\$}_{A} & = & \left(c-d\right)[\left(1-\frac{1}{e^{At}}\right)\left(\frac{1}{2}-p\right)\cos\left(\gamma \right)+\frac{\cos\left(2\beta +2\omega t\right)\sin^{2}\left(\gamma \right)}{2e^{Gt}}+\frac{\cos^{2}\left(\gamma \right)}{2e^{At}}] \end{array}$$ (34)

$$\begin{array}{ccc} \Delta {\$}_{AB} & = & \left(c-d\right)[\left(1-\frac{1}{e^{A_{A}t}}\right)\left(\frac{1}{2}-p_{A}\right)\cos\left(\gamma \right)+\frac{\cos\left(2\beta +2\omega t\right)\sin^{2}\left(\gamma \right)}{2e^{G_{A}t+G_{B}t}}+\frac{\cos^{2}\left(\gamma \right)}{2e^{A_{A}t}}]\;. \end{array}$$ (35)

For qualitative predictions of payoff’s character particularly important is the long time behavior of the above presented formulas. We consider $\Delta {\$}_{X}^{\infty }:={\text{lim}}_{t\to \infty }\Delta {\$}_{X}$ where *X* = *A*, *B*, *AB* i.e. we assume that the payoffs are calculated at the time significantly longer than the time of thermal equilibration of the player’s qubit. Explicit formulas (with no restriction imposed on *θ*) read as follows:

$$\begin{array}{ccc} \Delta {\$}_{B}^{\infty } & = & \frac{1}{2}\left(c-d\right)\left[\cos\left(\gamma \right)+2\left(p-\frac{1}{2}\right)\cos\left(\theta \right)\right]\cos\left(\gamma \right) \end{array}$$ (36)

$$\begin{array}{ccc} \Delta {\$}_{A}^{\infty } & = & \left(c-d\right)\left[\frac{1}{2}-\frac{\cos(\theta )\cos(\gamma )}{2}-p\right]\cos\left(\gamma \right) \end{array}$$ (37)

$$\begin{array}{ccc} \Delta {\$}_{AB}^{\infty } & = & \left(c-d\right)\left[\frac{1}{2}-p_{A}+\left(p_{B}-\frac{1}{2}\right)\cos\left(\theta \right)\right]\cos\left(\gamma \right) \end{array}$$ (38)

Explicit formulas for remaining strategies are postponed to the Appendix. There is a non-trivial issue factorizability of probabilities. We are aware of no reliable method to analyze such problem in simulations. The interested reader is referred to [17, 29, 30] for discussion of this problem.

## Results and Discussion

In this section we study one of best known examples of a game: the celebrated Prisoner Dilemma (PD) [1, 2]. Prisoner Dilemma is often used for analysis of various aspects of cooperation in economics, biology and network science [31]. The story says that two rational agents (prisoners) have to decide without communication whether cooperate or not. They might decide to not cooperate, even if it is obvious that they are better off if they do so. In its quantum version [4] the game ceases to be paradoxical for some classes of quantum strategies but we should stress here that the dilemma disappears due to dramatic enlargement of the set of strategies for both agents. Therefore, Quantum Prisoner Dilemma is a quite new game that reduces the classical one if the strategy sets are properly reduced. A general quantum game considered so far becomes reduced to the PD provided that parameters in payoffs Eq (28) fulfill the relation *c* > *a* > *b* > *d* [31]. We choose the following values:

$$\begin{array}{ccc} \left(a,b,c,d\right) & = & \left(3,1,5,0\right) \end{array}$$ (39)

Further we analyze in detail four strategies of players (or prisoners) assuming that one of them (Bob) can apply both classical and quantum strategies. Our aim is to present a relation between difference of Bob’s and Alice’s payoffs with respect to the entangling parameter *γ* in Eq (4). Initially we limit our attention to the case when there is only one noisy qubit belonging either to Bob or to Alice.

**Fig 1:**
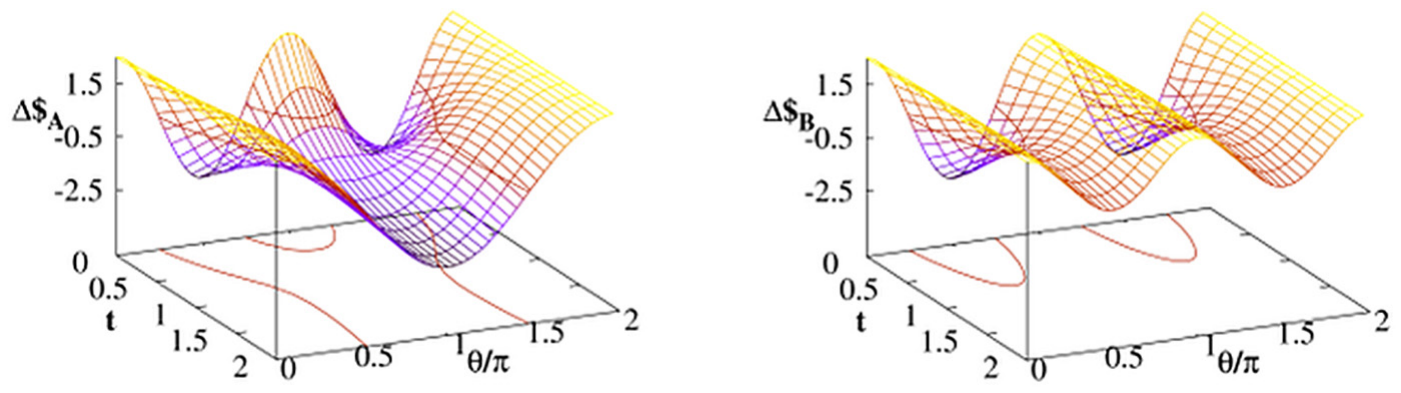
Payoff differences Eq (29) taken at different time instances *t* for Alice–Bob strategy profile (𝓘,𝓘*U*) with the quantum strategy Eq (12) with *U* = *U*(*π*/2,0, *π*/2). The thermal Davies environment (with *A* = 2*G* = 2) influences only one player (either Bob or Alice) and *p* = 0. The contours denote the border between positive and negative payoff difference.

First we consider (𝓘,𝓘*U*) strategy profile. The payoff differences Eqs (31 and 30) calculated at different time instants are presented in Fig (1). The quantum part of the Bob’s strategy is chosen to be *U* = *U*(*π*/2,0, *π*/2). The payoff difference Δ$ can be either positive (Bob is winning) or negative depending on the value of *γ*. This dependence is strongly affected by thermal environment. Moreover, this dependence is very different in the case when the environment is attached either to Bob’s or Alice’s qubit. Let us notice that in the case when thermal environment affects Bob’ qubit his payoff is in the long time limit always larger than the payoff of Alice i.e. Δ$_*B*_ > 0 for all *γ*. It is not the case when the noisy qubit belongs to Alice. There is a range of *γ* when Δ$_*A*_ < 0 i.e. when $_*B*_ < $_*A*_. In other words, in the situation when Alice can control or choose *γ* and possesses noisy qubit is favorable if she tries to win or at least minimize her losses. Let us also notice that there are parameters *γ* < *π*/4 such that Δ$_*A*_ > Δ$_*B*_ > Δ$ and, simultaneously Δ$ < 0. This range of parameters is particularly favorable for Bob who wins *due to* the presence of thermal environment.

**Fig 2:**
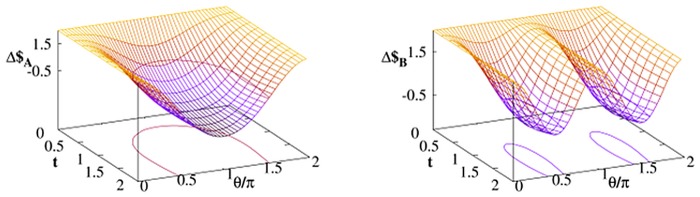
The same as in Fig (1) but for Alice–Bob strategy profile (𝓘,𝓕*U*).

As the second example we consider (𝓘,𝓕*U*) strategy profile. Here, in the absence of noise, the *γ*–dependence of payoff difference Δ$ is trivial: Bob always wins. In the presence of thermal bath it does not hold true any more. As presented in Fig (2), there exist *γ*’s resulting in Δ$_*A*_ < 0 i.e. Alice payoff becomes larger for sufficiently long time of interaction between her qubit and environment.

The payoffs for the remaining two strategy profiles (𝓕,𝓕*U*) and (𝓕,𝓘*U*) can be obtained form (𝓘,𝓘*U*) and (𝓘,𝓕*U*), respectively, by change of sign: Δ$ → −Δ$ and Δ$_*A*,*B*_ → −Δ$_*A*,*B*_. This symmetry is generic for PD game Eq (39) and does not depend on the specific choice of quantum part of Bob’s strategy *U*.

**Fig 3:**
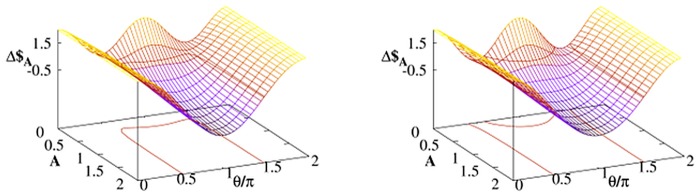
Payoff differences Eq (29) as a function of *A* calculated at time *t* = 2 for Alice–Bob strategy profiles (𝓘,𝓘*U*) (left panel) and (𝓘,𝓕*U*) (right panel) with the quantum strategy Eq (12) with *U* = *U*(*π*/2,0, *π*/2). The thermal Davies environment (with *G* = 1) influences only one player (either Bob or Alice) and *p* = 0.

**Fig 4:**
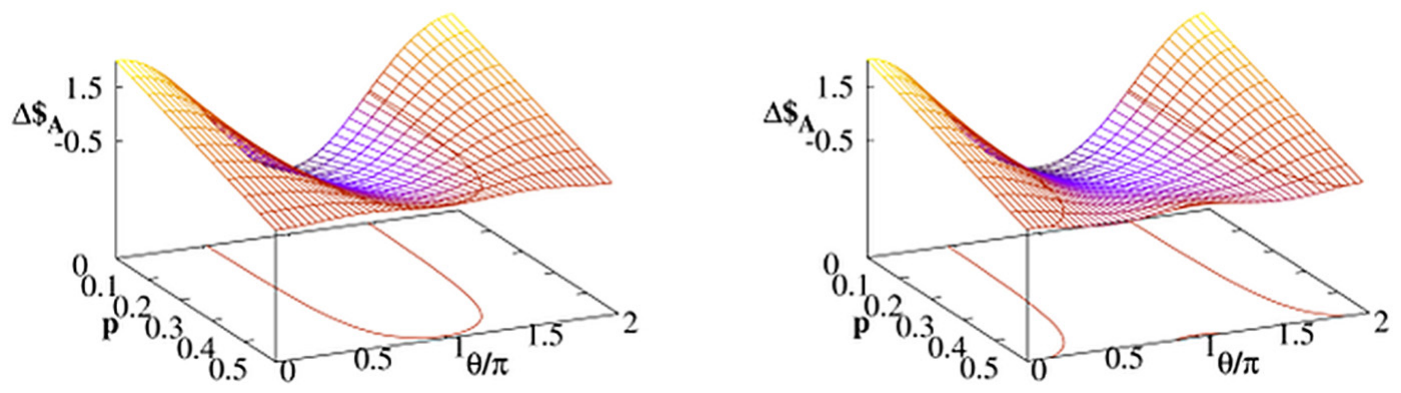
Payoff differences Eq (29) as a function of temperature *p* calculated at a time *t* = 2 for Alice–Bob strategy profiles (𝓘,𝓘*U*) (left panel) and (𝓘,𝓕*U*) (right panel) with the quantum strategy Eq (12) with *U* = *U*(*π*/2,0, *π*/2). The thermal Davies environment (with *G* = 1) influences only one player (either Bob or Alice) and *A* = 2.

The parameter *γ* is not the only one which affects Alice’s chance to win with Bob. We consider the case when the noisy qubit belongs to Alice. The energy relaxation parametrized by *A* is one of the parameters which most significantly affect character of thermal dissipation. As it was discussed in previous section for *A* = 0 (i.e. when there is only pure decoherence with no energy dissipation) Δ$_*A*_ = Δ$_*B*_ and the Bob’s ‘quantum benefit’ becomes neutralized. The larger *A* is the more different are the payoffs of Bob and Alice as presented for two strategies in Fig (3). The effect of increasing temperature is visualized in Fig (4). In the limit of high temperature Δ$_*A*_ is small but *positive*. In other words, for some strategies and for given *γ* Alice defeat Bob by warming her qubit.

**Fig 5:**
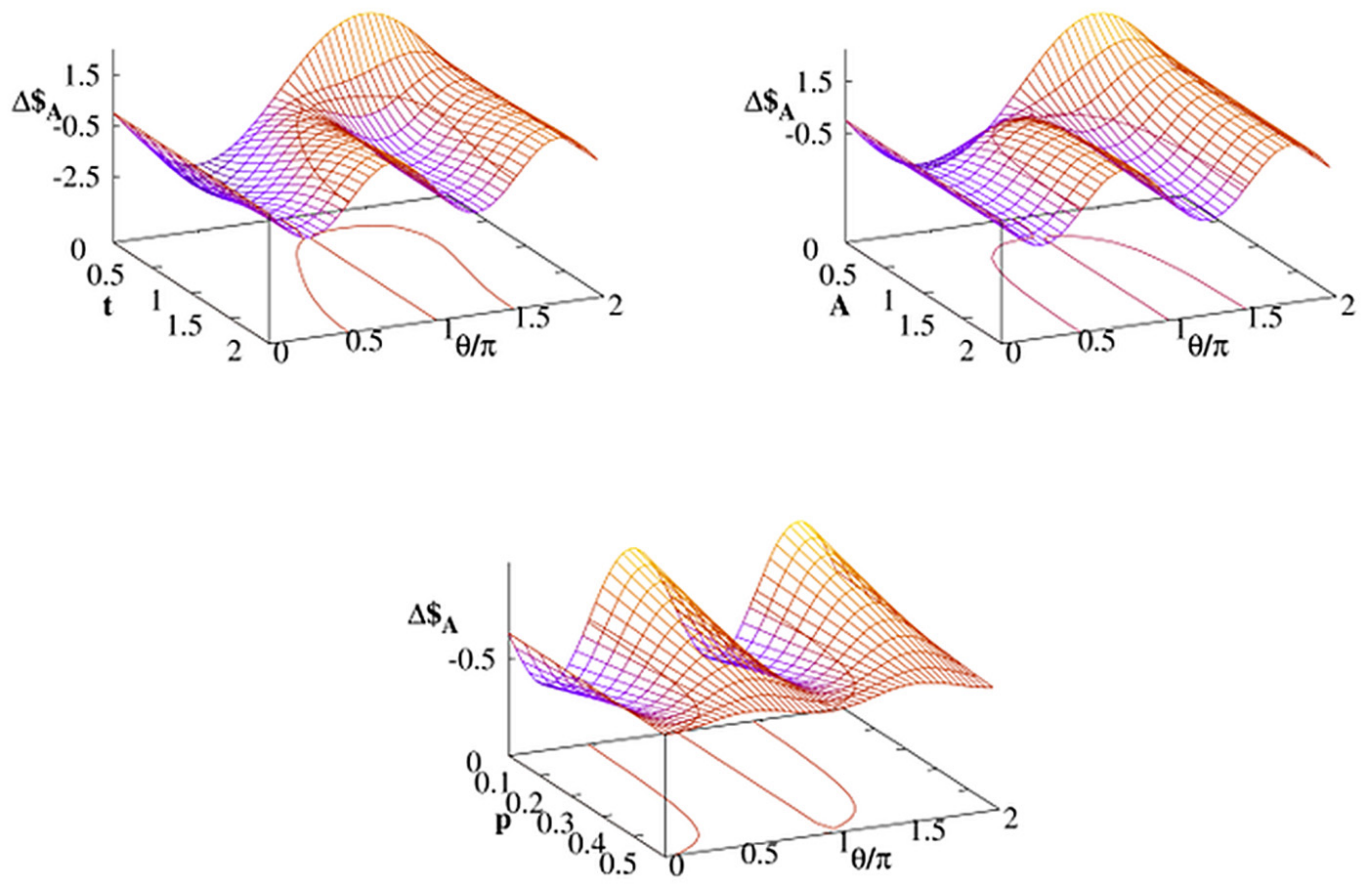
Payoff differences Eq (29) as a function of *A* calculated at time *t* = 2 for a mixed Alice–Bob strategy profile (𝓥_*c*_,𝓥_*c*_ *U*) with *V*_*c*_ = 𝓥/2 + 𝓕/2 and the quantum strategy Eq (12) with *U* = *U*(*π*/2,0, *π*/2). The thermal Davies environment (with *G* = 1) influences only Alice’s qubit. We set *A* = 0 and *p* = 0 in panels where these parameters are fixed.

The parameter *A* can influence payoffs in the games when the classical part of strategy is given by *V*_*c*_ = 𝓘/2+𝓕/2 i.e. for the game averaged with respect to both classical strategies. The results presented in Fig (5) indicate two basic features. First, after averaging Δ$_*A*_ = Δ$_*B*_. Second, changing *A* results in changing *γ*-’periodicity’ of payoffs.

The temperature dependence of payoffs is well visible in the long time limit (*t* → ∞) of the payoff differences Eq (29) here calculated for (𝓘,𝓘*U*) strategy:

$$\begin{array}{ccc} \Delta {\$}_{A} & = & -\frac{5}{4}\cos\left(\theta \right)+\left(\frac{5}{2}-5p\right)\cos\left(\gamma \right)-\frac{5}{8}\left[\cos\left(2\gamma +\theta \right)+\cos\left(-2\gamma +\theta \right)\right]\\ \Delta {\$}_{B} & = & \frac{5}{2}p\left[\cos\left(-\gamma +\theta \right)+\cos\left(\gamma +\theta \right)\right]-\frac{5}{4}[\cos\left(\gamma +\theta \right)-\cos\left(-\gamma +\theta \right)+\frac{5}{4}(1+\cos\left(2\gamma \right)\\ \Delta {\$}_{AB} & = & \frac{5}{2}p_{B}\left[\cos\left(\gamma +\theta \right)+\cos\left(-\gamma +\theta \right)\right]-\frac{5}{4}\left[\cos\left(\gamma +\theta \right)-\cos\left(-\gamma +\theta \right)\right]-\left(5p_{A}+\frac{5}{2}\right)\cos\left(\gamma \right) \end{array}$$ (40)

Formulas for different pure strategies can be obtained via the sign symmetry discussed above. Let us notice that for *θ* = *π*/2 (the strategy chosen in examples above) neither Δ$_*B*_ nor Δ$_*AB*_ depends on the temperature of Bob’s environment.

## Conclusions

A method of taking account of decoherence in quantum game theory has been presented. We have assumed that the interaction between qubits and their environments are weak and satisfy requirements for applying Davies weak coupling approach to reduced dynamics [21]. Actually, we have represented decoherence via Davies maps. Our analysis shows that the dependence is strongly affected by a thermal environment. The temperature dependence of payoffs is noticeable in the long time limit. Moreover, the presented analysis stresses that the payoffs can vary dramatically in cases when the environment is attached either to Bob’s or Alice’s qubit. This effect can be beneficial for one of the players as presented graphically for various special cases of payoff differences. It would be of great interest to adapt this approach to quantum games on networks of agents [32–34] because systems involving a large number of simple variables with mutual interactions appear frequently in various fields of research.

## Appendix: Explicit payoffs formulas

Here we provide explicit formulas for payoffs for three remaining quantum strategies (𝓘,𝓕*U*), (𝓕,𝓘*U*) and (𝓕,𝓕*U*) with *θ* = *π*/2 together with their long time limits:

$$\begin{array}{ccc} \Delta {\$}_{X}^{\infty } & = & \lim_{t\to \infty }\Delta {\$}_{X},\;\;X=A,B,AB \end{array}$$ (41)

calculated for an arbitrary value of *θ*.

## (𝓘,𝓕*U*)

$$\begin{array}{ccc} \Delta {\$}_{B} & = & \frac{1}{2}\left(c-d\right)\left[\cos^{2}\left(\gamma \right)+\frac{\sin^{2}\left(\gamma \right)\cos\left(2\alpha -2\omega t\right)}{e^{Gt}}\right] \end{array}$$ (42)

$$\begin{array}{ccc} \Delta {\$}_{A} & = & \left(c-d\right)[\left(1-\frac{1}{e^{At}}\right)\left(\frac{1}{2}-p\right)\cos\left(\gamma \right)+\frac{\cos\left(2\alpha -2\omega t\right)\sin^{2}\left(\gamma \right)}{2e^{Gt}}+\frac{\cos^{2}\left(\gamma \right)}{2e^{At}}] \end{array}$$ (43)

$$\begin{array}{ccc} \Delta {\$}_{AB} & = & \left(c-d\right)[\left(1-\frac{1}{e^{A_{A}t}}\right)\left(\frac{1}{2}-p_{A}\right)\cos\gamma +\frac{\cos\left(2\alpha -2\omega t\right)\sin^{2}\left(\gamma \right)}{2e^{G_{A}t+G_{B}t}}+\frac{\cos^{2}\left(\gamma \right)}{2e^{A_{A}t}}] \end{array}$$ (44)

$$\begin{array}{ccc} \Delta {\$}_{B}^{\infty } & = & \frac{1}{2}\left(c-d\right)\left[\cos\left(\gamma \right)-2\left(p-\frac{1}{2}\right)\cos\left(\theta \right)\right]\cos\left(\gamma \right) \end{array}$$ (45)

$$\begin{array}{ccc} \Delta {\$}_{A}^{\infty } & = & \left(c-d\right)\left[\frac{1}{2}+\frac{\cos(\theta )\cos(\gamma )}{2}-p\right]\cos\left(\gamma \right) \end{array}$$ (46)

$$\begin{array}{ccc} \Delta {\$}_{AB}^{\infty } & = & \left(c-d\right)\left[\frac{1}{2}-p_{A}-\left(p_{B}-\frac{1}{2}\right)\cos\left(\theta \right)\right]\cos\left(\gamma \right) \end{array}$$ (47)

## (𝓕,𝓘*U*)

$$\begin{array}{ccc} \Delta {\$}_{B} & = & \frac{1}{2}\left(d-c\right)\left[\cos^{2}\left(\gamma \right)+\frac{\sin^{2}\left(\gamma \right)\cos\left(2\alpha -2\omega t\right)}{e^{Gt}}\right] \end{array}$$ (48)

$$\begin{array}{ccc} \Delta {\$}_{A} & = & \left(d-c\right)[\left(1-\frac{1}{e^{At}}\right)\left(\frac{1}{2}-p\right)\cos\left(\gamma \right)+\frac{\cos\left(2\alpha -2\omega t\right)\sin^{2}\left(\gamma \right)}{2e^{Gt}}+\frac{\cos^{2}\left(\gamma \right)}{2e^{At}}] \end{array}$$ (49)

$$\begin{array}{ccc} \Delta {\$}_{AB} & = & \left(d-c\right)[\left(1-\frac{1}{e^{A_{A}t}}\right)\left(\frac{1}{2}-p_{A}\right)\cos\gamma +\frac{\cos\left(2\alpha -2\omega t\right)\sin^{2}\left(\gamma \right)}{2e^{G_{A}t+G_{B}t}}+\frac{\cos^{2}\left(\gamma \right)}{2e^{A_{A}t}}] \end{array}$$ (50)

$$\begin{array}{ccc} \Delta {\$}_{B}^{\infty } & = & \frac{1}{2}\left(d-c\right)\left[\cos\left(\gamma \right)-2\left(p-\frac{1}{2}\right)\cos\left(\theta \right)\right]\cos\left(\gamma \right) \end{array}$$ (51)

$$\begin{array}{ccc} \Delta {\$}_{A}^{\infty } & = & \left(d-c\right)\left[\frac{1}{2}+\frac{\cos(\theta )\cos(\gamma )}{2}-p\right]\cos\left(\gamma \right) \end{array}$$ (52)

$$\begin{array}{ccc} \Delta {\$}_{AB}^{\infty } & = & \left(d-c\right)\left[\frac{1}{2}-p_{A}-\left(p_{B}-\frac{1}{2}\right)\cos\left(\theta \right)\right]\cos\left(\gamma \right) \end{array}$$ (53)

## (𝓕,𝓕*U*)

$$\begin{array}{ccc} \Delta {\$}_{B} & = & \frac{1}{2}\left(d-c\right)\left[\cos^{2}\left(\gamma \right)+\frac{\cos\left(2\beta +2\omega t\right)\sin^{2}\left(\gamma \right)}{e^{Gt}}\right] \end{array}$$ (54)

$$\begin{array}{ccc} \Delta {\$}_{A} & = & \left(d-c\right)[\left(1-\frac{1}{e^{At}}\right)\left(\frac{1}{2}-p\right)\cos\left(\gamma \right)+\frac{\cos\left(2\beta +2\omega t\right)\sin^{2}\left(\gamma \right)}{2e^{Gt}}+\frac{\cos^{2}\left(\gamma \right)}{2e^{At}}] \end{array}$$ (55)

$$\begin{array}{ccc} \Delta {\$}_{AB} & = & \left(d-c\right)[\left(1-\frac{1}{e^{A_{A}t}}\right)\left(\frac{1}{2}-p_{A}\right)\cos\left(\gamma \right)+\frac{\cos\left(2\beta +2\omega t\right)\sin^{2}\left(\gamma \right)}{2e^{G_{A}t+G_{B}t}}+\frac{\cos^{2}\left(\gamma \right)}{2e^{A_{A}t}}] \end{array}$$ (56)

$$\begin{array}{ccc} \Delta {\$}_{B}^{\infty } & = & \frac{1}{2}\left(d-c\right)\left[\cos\left(\gamma \right)+2\left(p-\frac{1}{2}\right)\cos\left(\theta \right)\right]\cos\left(\gamma \right) \end{array}$$ (57)

$$\begin{array}{ccc} \Delta {\$}_{A}^{\infty } & = & \left(d-c\right)\left[\frac{1}{2}-\frac{\cos(\theta )\cos(\gamma )}{2}-p\right]\cos\left(\gamma \right) \end{array}$$ (58)

$$\begin{array}{ccc} \Delta {\$}_{AB}^{\infty } & = & \left(d-c\right)\left[\frac{1}{2}-p_{A}+\left(p_{B}-\frac{1}{2}\right)\cos\left(\theta \right)\right]\cos\left(\gamma \right) \end{array}$$ (59)
